# Five‐year evaluation of linear accelerator‐based SRS platform isocentricity

**DOI:** 10.1002/acm2.14597

**Published:** 2024-12-02

**Authors:** Yohan A. Walter, Anne N. Hubbard, Phillip F. Durham, Hsinshun T. Wu

**Affiliations:** ^1^ Department of Radiation Oncology Willis Knighton Cancer Center Shreveport Louisiana USA

**Keywords:** linear accelerator, QA, quality assurance, SRS, SRT, stereotactic radiosurgery

## Abstract

Linear accelerator (LINAC)‐based stereotactic radiosurgery (SRS) has become a mainstay in the management of intracranial tumors. However, the high fractional doses and sharp gradients used in SRS place heavy demands on geometric accuracy. Image guidance systems such as ExacTrac (ETX, Brainlab AG, Munich, Germany) have been developed to facilitate position verification at nonzero table angles. Though convenient, potential loss of mechanical rigidity between the imaging and treatment systems can be cause for concern, as the ETX system is not mounted to the rotating gantry. In this retrospective study, we analyzed 518 Winston‐Lutz (WL) tests performed in the last 5 years with ETX alignment on our Elekta Versa HD (Elekta AB, Stockholm, Sweden) linear accelerator to determine the achievable limits of precision and stability over time for our LINAC‐based SRS platform. Results demonstrated remarkable stability over time. 3D and directional misalignments never exceeded 1.0 mm over the study period; however, table rotation was shown to be the most significant source of positional uncertainty. Gantry sag, as measured by gun‐to‐target misalignments at the gantry‐0 and gantry‐180‐degree positions, was consistent, measuring 1.23 ± 0.18 mm over the study period. Measured accuracy was well within acceptable tolerances for cranial SRS treatment delivery. Notably, the use of the ETX system for intrafraction repositioning effectively eliminates couch walkout, the most significant source of uncertainty identified in this study. Our results thus corroborate safe SRS treatment delivery on our Versa HD with ExacTrac image guidance.

## 1 INTRODUCTION

Agreement between mechanical, dosimetric, and imaging isocenters is critical for safe delivery of radiotherapy procedures, particularly for those treated under stereotaxis.^1^, ^2^, ^3^, ^4^ Brainlab ExacTrac (ETX, Brainlab AG, Munich, Germany) is a kilovoltage planar oblique imaging system which offers fast positioning and verification imaging at all table angles with minimal additional x‐ray dose. ETX imaging panels are mounted to the vault ceiling, with x‐ray tubes mounted on or under the floor, rather than both components being mounted on the rotating gantry. Though this arrangement eliminates moving parts and associated uncertainties, the inherent mechanical rigidity between the imaging and treatment delivery systems does not exist, as compared to on‐board imaging (OBI) systems such as cone beam computed tomography (CBCT). The overall performance and accuracy of the ETX system has been evaluated in several studies.^5^, ^6^, ^7^, ^8^

The Winston‐Lutz (WL) test represents the gold standard in the verification of alignment between the radiation, mechanical, and imaging isocenters. To this end, the WL test and its variants have become a staple in most quality assurance (QA) regimens,^2^, ^4^ particularly for linear accelerator‐based stereotactic radiosurgery (SRS) programs, since the steep dose gradients and high biological effective doses used in SRS place heavy demands on end‐to‐end geometric accuracy.

In developing an SRS program, users have a broad range of platforms to choose from. Significant factors in the decision between platforms include projected long‐term stability, expected maintenance, and achievable delivery accuracy. Since the WL test evaluates mechanical, imaging, and radiation isocenter congruence, long‐term WL data can be demonstrative of end‐to‐end performance and stability over time. This information can be used to develop QA tolerances, action levels, and identify potential failure modes that lead to geometric uncertainty in treatment delivery.

The need for quantitative long‐term machine performance data has been established in the literature. For example, Gao and Anand recently performed off‐isocenter Winston‐Lutz‐Gao tests on seven Varian linear accelerators (Varian Medical Systems, Palo Alto, CA, USA) which demonstrated variations in results that appeared to correlate with machine age.^9^ Though other studies reported WL test results for several models of Varian linear accelerator over extended periods,^10^, ^11^ there is a paucity of data reported for the same system over periods greater than 1 year. Furthermore, there is little long‐term data available in the literature for Elekta linear accelerators. Since Elekta units (Elekta AB, Stockholm, Sweden) are known to have accentuated gantry sag effects compared to competitor systems,^1^, ^3^, ^12^, ^13^ which presents a significant potential concern for SRS treatment delivery, there is a need for long‐term data to determine the achievable mechanical precision and to characterize behavior with increasing machine age for Elekta systems.

The purpose of this study was therefore to evaluate the long‐term isocentricity between the ExacTrac image‐guided radiotherapy (IGRT) system and an Elekta Versa HD linear accelerator using routine WL tests performed over a 5‐year period. To our knowledge, this study is the first to report long‐term WL results for an Elekta linear accelerator.

## 2 METHODS

### 2.1 Equipment

Our Elekta Versa HD was commissioned in 2016. The unit is equipped with the 6‐degree‐of‐freedom (DoF) HexaPOD couch and a 160‐Multileaf collimator (MLC) Agility treatment head. Individual MLC leaves have a constant width of 5.0 mm projected at the isocenter. The system is also equipped with amorphous silicon electronic portal (EPID) and kV imaging panels.

The ExacTrac system features two ceiling‐mounted imaging panels and two paired floor‐mounted kilovoltage x‐ray tubes. During the treatment delivery, ETX was used for all initial patient positioning and intrafraction repositioning at each table angle. The system features both kilovoltage x‐ray imaging and infrared fiducial tracking for real‐time position monitoring.

A WL test was performed daily prior to each ExacTrac‐guided SRS or fractionated stereotactic radiotherapy (SRT) procedure over the last 5 years. These cases included both MLC‐based and cone‐based functional treatments.

WL tests used either a standard cube phantom with an 8.0 mm metal ball bearing (BB) at its center, or the Multi‐Met phantom (Sun Nuclear Corporation, Melbourne, FL, USA), a cuboid with multiple 5.0 mm diameter BBs at several positions in the phantom. In this study, only results for the BB placed at the imaging isocenter were included.

### 2.2 ETX isocenter calibration

The ETX imaging isocenter was calibrated relative to the radiation beam using a cube phantom with an 8.0 mm metal BB and portal imaging at the four cardinal gantry angles to place the target at the radiation isocenter. To account for gantry sag effects, the radiation isocenter was placed such that the gun‐to‐target (G‐T) offset was evenly split between the gantry‐0 (G0) and gantry‐180‐degree (G180) positions. This position was then defined as the ETX imaging isocenter.

### 2.3 Phantom setup and measurement

The WL phantom was placed at the imaging isocenter using ETX and the HexaPOD table. The tolerance for initial cube positioning was a residual translational shift of ≤0.10 mm in any direction as determined by ExacTrac X‐ray imaging.

The tested fields are listed in Table 1 and were selected to interrogate clinically used gantry, collimator, and table angles for cranial SRS/SRT. All fields used a 6 MV flattening filter free (FFF) beam with a 3 cm × 3 cm aperture for the cube phantom, a 2 cm × 2 cm aperture for the Multi‐Met phantom, or a 1.7 cm‐diameter stereotactic cone (Aktina Medical, Congers, NY, USA).

**TABLE 1:** Field sets used in Winston‐Lutz tests.

MLC or Cone, 8.0 mm BB (*N* = 361)			MLC, 5.0 mm BB (*N* = 157)		
Gantry [deg]	Collimator [deg]	Table [deg]	Gantry [deg]	Collimator [deg]	Table [deg]
0	0	0	0	0	0
90	0	0	90	0	0
150	0	0	150	0	0
180	0	0	180	0	0
270	0	0	270	0	0
330	0	0	330	0	0
0	90	0	0	90	0
0	270	0	0	270	0
0	0	30	0	45	45
0	0	90	0	0	90
0	0	270	0	0	270
0	0	330	0	45	315

Abbreviation: MLC, multileaf collimator.

Per our department protocol, during treatment delivery, ETX images were taken at every couch angle and if needed, repositioning shifts were applied. Therefore, in consideration of on‐treatment corrections, the full WL test with couch rotations was only performed for the first delivered fraction of the week for a given patient plan. Measurements at nonzero couch angles were foregone for all other fractions in the treatment course.

### 2.4 Data recording and analysis

Results of 518 WL tests performed between 2019 and 2024 were compiled for analysis. Three hundred and seventy‐one of the tests included all twelve tested fields, while the remaining 147 only included gantry and collimator rotations. Twenty‐four of the recorded tests were performed with the 1.7 cm stereotactic cone.

Portal images were loaded into the RIT Complete version 6.5.64 3D EPID Stereotactic Alignment module (Radiological Imaging Technology, Inc., Colorado Springs, CO, USA). The RIT system compared the metal marker position relative to the field aperture and reported measured misalignment in the IEC X, Y, and Z‐planes, as well as the total 3D misalignment. The maximum single field displacement (max SFD) was taken as the magnitude of the largest measured offset for the portal image with the greatest misalignment taken in each test. The gantry, collimator, and table angle combination which resulted in the max SFD for each WL test were recorded. Gantry, collimator, and table virtual starshots were calculated in RIT software based on measured angles. The starshot radius was recorded for analysis. Gantry sag was measured by subtracting the G‐T offset measured at G180 from that measured at the G0 position for each test.

Statistical analysis was performed in OriginPro (OriginLab, Northampton, MA, USA). Analysis of variance was used to determine differences between means at the *p* < 0.05 significance level.

## 3 RESULTS

### 3.1 Isocenter alignment and displacement

IEC X, Y, Z, and 3D displacements are listed in Table 2. All 518 tests yielded submillimeter isocenter deviations in the IEC X, Y, and Z directions. All 3D displacements were submillimeter. One hundred and thirty tests (25.1%) had 3D displacements ≥0.5 mm and 16 tests (3.1%) had 3D displacement ≥0.75 mm. The overall average 3D displacement across all tests in the study period was 0.39 ± 0.17 mm.

**TABLE 2:** Recorded Winston‐Lutz test results grouped by year during the study period.

	Year 1 (*N* = 109)	Year 2 (*N* = 77)	Year 3 (*N* = 89)	Year 4 (*N* = 116)	Year 5 (*N* = 127)
Avg dX [mm] (SD)	0.32 (0.10)	0.11 (0.22)	0.00 (0.11)	0.16 (0.11)	0.15 (0.18)
Avg dY [mm] (SD)	−0.13 (0.16)	−0.14 (0.17)	−0.14 (0.11)	−0.05 (0.10)	−0.10 (0.15)
Avg dZ [mm] (SD)	0.35 (0.30)	0.46 (0.19)	0.16 (0.21)	0.18 (0.15)	0.05 (0.56)
Avg 3D [mm] (SD)	0.51 (0.14)	0.57 (0.15)	0.30 (0.15)	0.31 (0.11)	0.33 (0.11)
Avg Max SFD [mm] (SD)	0.93 (0.15)	1.22 (0.33)	0.80 (0.11)	0.79 (0.10)	0.87 (0.17)
Avg Max SFD, Excl. Table Kicks [mm] (SD)	0.87 (0.10)	0.86 (0.09)	0.80 (0.11)	0.79 (0.10)	0.81 (0.11)
Gantry Sag [mm] (SD)	1.25 (0.12)	1.24 (0.17)	1.26 (0.14)	1.22 (0.21)	1.21 (0.23)
Gantry Starshot Radius [mm] (SD)	0.26 (0.10)	0.32 (0.08)	0.50 (0.11)	0.25 (0.09)	0.30 (0.10)
Table Starshot Radius [mm] (SD)	0.36 (0.17)	0.79 (0.30)	0.31 (0.11)	0.33 (0.11)	0.40 (0.15)
Collimator Starshot Radius [mm] (SD)	0.25 (0.16)	0.60 (0.42)	0.20 (0.10)	0.20 (0.11)	0.27 (0.19)

Abbreviations: dX, dY, dZ, displacement in IEC X, Y, and Z directions; Max SFD, maximum single field displacement; SD, standard deviation.

The maximum single field displacement averaged 0.91 ± 0.23 mm in magnitude. Table rotation was associated with the largest maximum SFDs (*p* < 0.001). Excluding cases for which table kicks were the source of the max SFD, which simulates executed repositioning at each table angle, the average dropped to 0.82 ± 0.11 mm.

**FIGURE 1:**
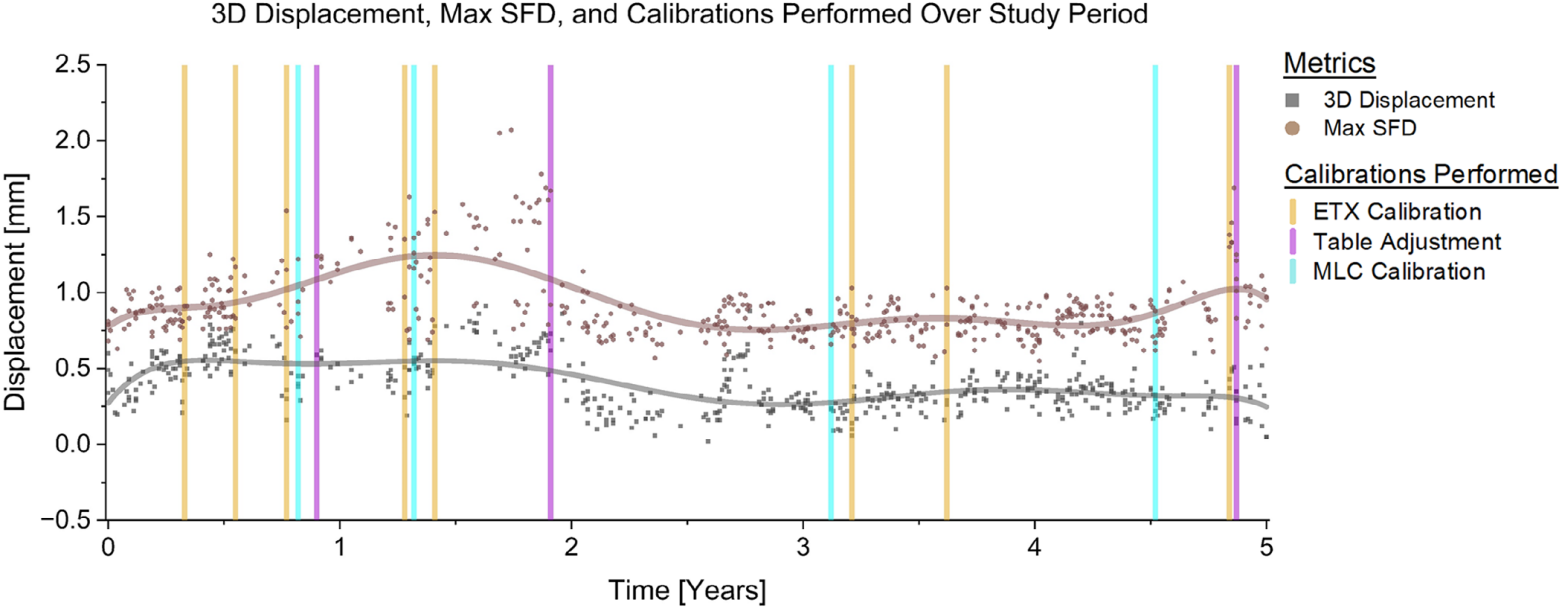
3D displacement and max SFD over the 5‐year period. Calibrations were performed on dates indicated by vertical markers. ETX, ExacTrac, MLC, multileaf collimator; SFD, single field displacement.

**FIGURE 2:**
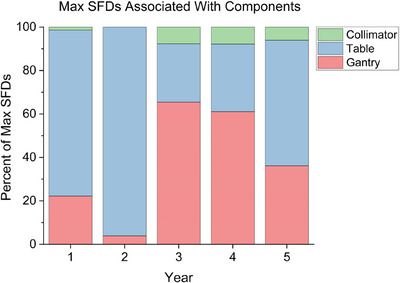
Percentage of maximum single field displacements associated with tested machine components by year. Only full WL tests including couch rotations were considered.

We observed significant table walkout issues in the first, second, and fifth years of the study period, which required mechanical adjustment (Figures 1 and 2). Excluding cases where table walkout was the most significant source of error, thus significantly changed the max SFD in those years (*p* < 0.05), particularly in year 2 (Table 2, Figure S1).

The max SFD was associated with table kicks in 51.2% of cases for which table angles were tested. 71.4%, 96.1%, 21.2%, 28.2%, and 52.2% of the max SFDs in years 1–5, respectively, were attributed to table rotation. Gantry rotation was the second most prevalent factor, accounting for 31.2%, 31.2%, 53.9%, 54.3%, and 40.9% of max SFDs across all cases in each year, respectively (Figure 2).

### 3.2 Gantry sag

**FIGURE 3:**
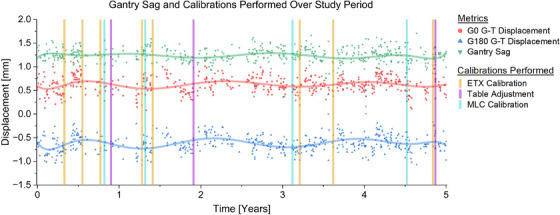
Gun‐to‐Target (G‐T) deviations measured at Gantry‐0 degrees (G0) and Gantry‐180 degrees (G180) over the study period. Gantry sag was taken as the difference between the G‐T deviations at G0 and G180. Calibrations were performed on dates indicated by vertical markers.

Gantry sag, as determined by the difference in G‐T alignment at G0 and G180 positions, was 1.23 ± 0.18 mm averaged across all tests. There was no significant difference in measured gantry sag by year (Table 2, Figure 3).

### 3.3 Virtual starshots

The average radii for gantry, couch, and collimator starshots were 0.32 ± 0.13 mm, 0.41 ± 0.23 mm, and 0.28 ± 0.25 mm, respectively. The table starshot had the largest radius in all but year 3, while the gantry had the largest starshot radius (*p* < 0.05).

## 4 DISCUSSION

A total of 40–60 patients are treated annually with ExacTrac‐guided SRS/SRT at Willis Knighton Cancer Center (Shreveport, LA, USA). Ensuring the stability of the mechanical, radiation, and imaging isocenters is therefore paramount for continued safe and effective LINAC‐based SRS/SRT delivery. In this study, we observed isocenter congruency results over an extended period to determine the overall mechanical stability of our imaging and treatment delivery systems, as well as to observe trends with the machine age. Results included both MLC‐based and cone‐based WL tests which used ExacTrac image guidance for initial phantom positioning.

**FIGURE 4:**
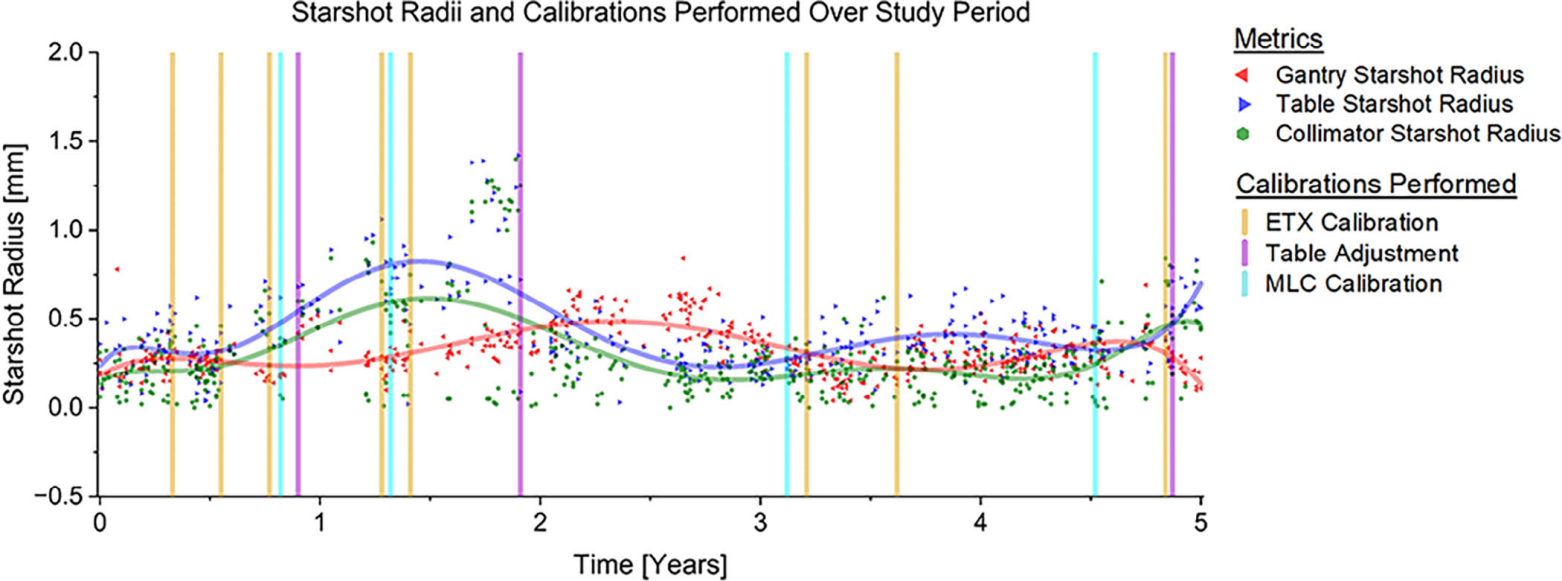
Virtual Starshot Radii for machine components based on tested fields as measured in RIT software over the study period. Calibrations were performed on dates indicated by vertical markers.

We observed drifts in measured offsets over the 5‐year study period. However, significant changes in time were only observed when prompt adjustments were not performed, such as in years 1–2, where the max SFD (Figure 1) and starshot radius (Figure 4) climbed steadily until the table isocenter was adjusted. When table rotations were excluded (Figure S1), there was no observed trend toward worsening alignment over the study period, indicating the stability of our combined system with time. The system retained a stable submillimeter 3D alignment over the study period, with 3D offsets never exceeding 1.0 mm. The system thus maintained isocenter congruence within acceptable limits for SRS delivery.^2^, ^14^

### 4.1 Sources of misalignment and table walkout

While the 3D displacement more strongly correlates with ETX and treatment beam isocenter agreement, the SFD allows for the evaluation of individual machine component alignment. The maximum SFD was used to identify combinations of gantry, collimator, and table rotations at which agreement between the imaging and radiation isocenters was worst.

Overall, table rotations were associated with the largest SFD magnitude (*p* < 0.001) and accounted for the greatest percentage of max SFDs (Figure 2, Figure S2). Additionally, as seen in the first 2 years, the max SFD and table starshot radius tended to increase in magnitude over time when interventions were not performed promptly (Figures 1 and 4).

When table rotations were performed, maximum SFDs were associated with table kicks in 51.2% of cases. The couch had the largest virtual starshot radius in all but year 3 of the study as compared to the gantry or collimator (*p* < 0.05). Table walkout was thus the greatest source of misalignment, which agrees with reports in the literature.^13^, ^15^As noted in Section 2.1, however, during patient treatment, the ETX system was used at each couch angle for verification imaging and repositioning, should table walkout or patient motion exceed repositioning tolerances. In contrast, the ETX system was only used for initial WL phantom alignment in this study. Therefore, the use of the ETX system at each couch angle during treatment effectively eliminates the most significant source of misalignment measured in this study.

### 4.2 Gantry sag

A comprehensive study by Rowshanfarzad et al. quantified gantry sag for nine Elekta units using a similar EPID‐based method as the one presented in this work.^16^ Their group reported that gantry sag was within 2 mm for all tested units, but the group also observed a correlation between gantry sag and machine age.^16^ A comparative study by Jursinic et al. reported a 1.2 mm gantry sag measured for their Elekta system, which was approximately three times the magnitude measured on systems from another vendor.^13^ The 1.23 ± 0.18 mm gantry sag measured in this study agrees with measurements previously reported in the literature.^1^, ^12^, ^13^, ^16^

Though the known gantry sag effects seen in Elekta units may be perceived as contraindicative of performing LINAC‐based SRS on these systems, our results demonstrated a durable gantry sag pattern (Table 2, Figure 3) which could be accounted for in ExacTrac isocenter calibration, as described in Section 2.2. By performing the IGRT isocenter calibration such that the isocenter position splits the sag‐induced offsets between measurements at G0 and G180, the overall measured sag could be reduced to two sub‐millimeter G‐T offsets at G0 and G180. Therefore, sub‐millimeter alignment accuracy is both achievable and stable for Elekta units when gantry sag is adequately accounted for in isocenter calibration.

### 4.3 Study limitations

Though the agreement between the ExacTrac and radiation isocenters was durable in this study, it is critical that routine isocenter verifications be performed, particularly in consideration of the lack of mechanical rigidity between the gantry and the room‐mounted imaging system. Furthermore, the ceiling‐mounted imaging panels were occasionally struck by patients or staff, prompting verification of WL tests and calibrations.

Our facility follows a preventative maintenance schedule which includes routine MLC and optical system calibrations, which adjusted the radiation field aperture and thus position relative to the metal BB in WL tests. These frequent checks and routine preventative maintenance, therefore, involved adjustments which likely influenced the measured stability of our system over time. Thus, for a system that is not adequately monitored, maintained, and routinely calibrated, users may observe more significant or compounding misalignment with increasing machine age. Notably, however, the ETX isocenter calibration was performed relatively infrequently over the study period. In total, 11 isocenter calibrations were performed, with 7 being done in the first year of the study period. Therefore, the alignment between ETX and radiation isocenter was stable with minimal routine upkeep.

A second limitation of this study is the known sensitivity of WL tests to initial phantom setup and, therefore, inter‐user variability. The tests reported in this study were performed by 11 different physicists and physics residents, which may have influenced measurements over time. However, the combination of image‐guidance and the associated ±0.10 mm initial phantom positioning tolerance likely significantly reduced this variability.

## 5 CONCLUSION

We have shown that an Elekta Versa HD paired with a Brainlab ExacTrac IGRT system can achieve and maintain sub‐millimeter agreement between imaging and radiation isocenters. Despite known gantry sag effects observed for various Elekta linear accelerators in the literature, our system maintains adequate precision for the safe delivery of stereotactic procedures. Our results also demonstrate that, provided users perform routine calibrations, the agreement between the ExacTrac system and the treatment beam isocenter is stable despite the IGRT system not being mounted directly to the rotating gantry. The ExacTrac system thus offers the combined benefit of imaging quickly and at any table angle, thereby reducing the influence of couch walkout on end‐to‐end positional misalignment.

Though our results corroborate safe stereotactic radiotherapy treatment delivery on our system, considering the increasing use of the single‐isocenter, multi‐target treatment delivery technique, further characterization for off‐isocenter targets and other accelerators may further benefit users in developing new or advancing existing stereotactic programs. Additionally, long‐term analysis of other performance parameters for modern machines, paired with effective failure modes and effects analysis (FMEA), may assist in evaluating or developing equipment‐specific tolerances and action levels for current systems.

## AUTHOR CONTRIBUTIONS

All authors made substantial contributions to the design, preparation, and execution of the presented study, including data acquisition, analysis, interpretation, manuscript drafting, and review.

## CONFLICT OF INTEREST STATEMENT

Y.A.W. and H.T.W. disclose an active research agreement with CQ Medical (Avondale, PA, USA). Y.A.W. reports receiving travel funding from CQ Medical.

## Supporting information



Supporting Information

## Data Availability

The data that support the findings of this study are available from the corresponding author upon reasonable request.
