# Supplementary material for: Five‐year evaluation of linear accelerator‐based SRS platform isocentricity
Source: J Appl Clin Med Phys. 2024 Dec 2;26(4):e14597. doi: 10.1002/acm2.14597 (PMC11969110; doi:10.1002/acm2.14597)
Supplement: Supplementary file 1 — Supporting Information [file ACM2-26-e14597-s001.docx]

Supplement Materials for Manuscript, “Five-Year Evaluation of Linear Accelerator-Based SRS System Isocentricity”


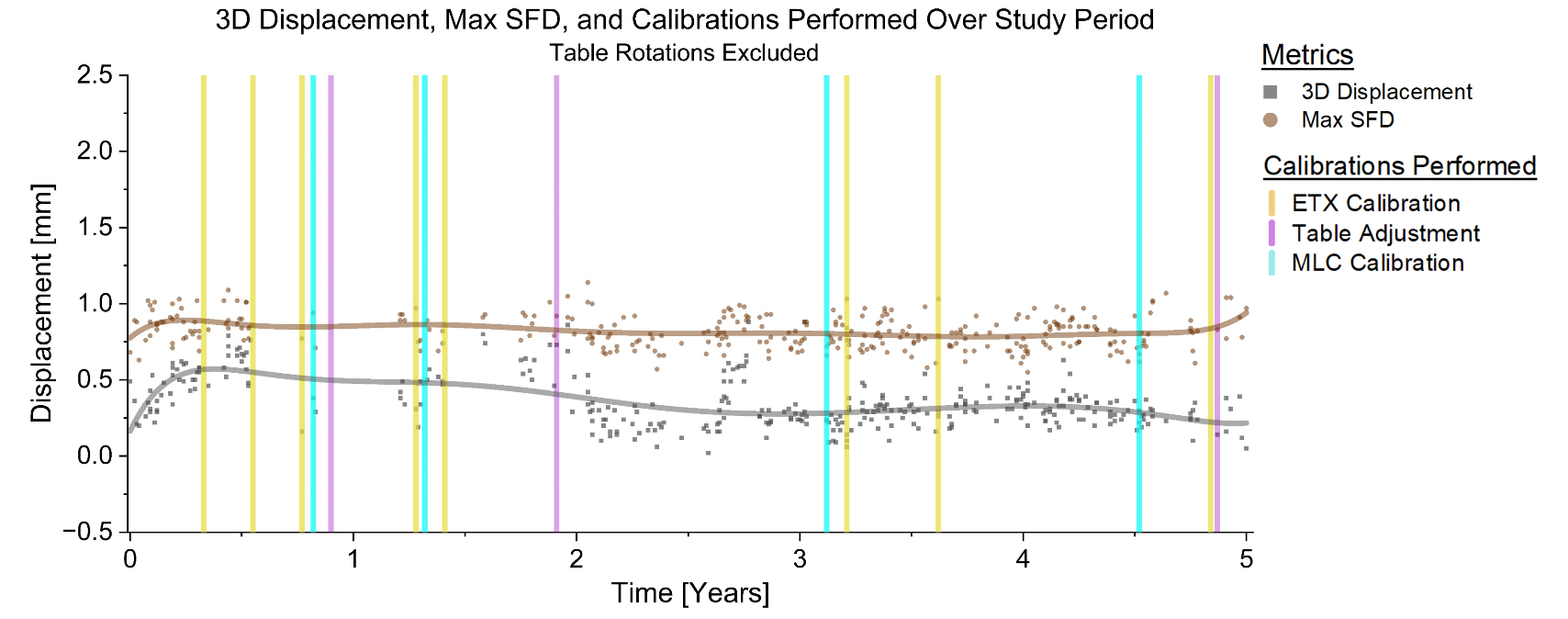


Figure S.1: 3D displacement and Max SFD over the five-year period with cases where table rotations yielded the maximum SFD excluded. Abbreviations: SFD = single field displacement, ETX = ExacTrac, MLC = multileaf collimator.

Figure S.1 shows the five-year trend of max SFD and 3D displacement with instances where the table was associated with the maximum SFD removed, demonstrating that the gantry and collimator were associated with overall stable maximum SFD and submillimeter 3D displacement over the study interval.


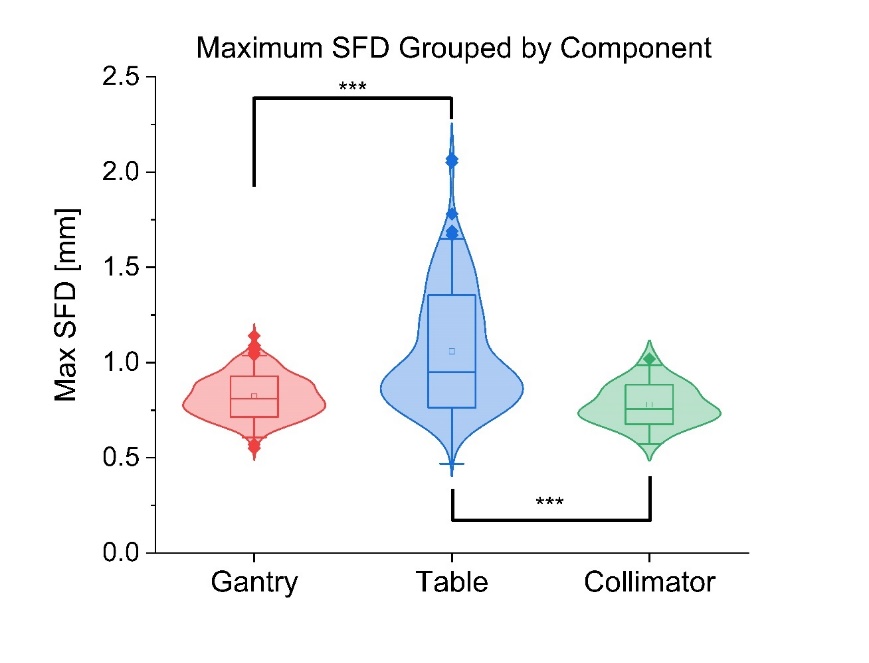


Figure S.2: Magnitude of maximum single field displacement (SFD) for cases where each component was associated with the max SFD (*** = p<0.001).

Figure S.2 shows the magnitude of the max SFD by component. As demonstrated, the treatment table was most frequently associated with the maximum SFD for a given test, and also yielded the largest magnitude of maximum single field displacements (p<0.001).
